# Effects of Experimental Conditions on Extraction Yield of Extracellular Polymeric Substances by Cation Exchange Resin

**DOI:** 10.1100/2012/751965

**Published:** 2012-08-01

**Authors:** Jinwoo Cho, Slawomir W. Hermanowicz, Jin Hur

**Affiliations:** ^1^Department of Environment & Energy, Sejong University, Seoul 143-747, Republic of Korea; ^2^Department of Civil and Environmental Engineering, University of California, Berkeley, CA 94720, USA

## Abstract

Effects of experimental conditions on the yield of extracellular polymeric substances (EPSs) extraction by cation exchange resin (CER) were investigated using activated sludge flocs. The experimental variables included resin dose, extraction time, sample dilution, and storage time. An empirical model was proposed to describe the kinetics of extraction process. The extraction yield increases with the extraction time and CER dose until it reached the maximum amount of EPS extraction. The maximum yield of EPS was affected as well by the sample dilution, exhibiting a decreasing trend with increasing dilution factor. It was also found that the amount of EPS extracted from a raw sample depends on the storage time. Once EPS was extracted from the sample, however, the EPS keeps its original quantity under storage at 4°C. Based on the model, the maximum amount of EPS extraction and yield rate could be estimated for different conditions. Comparing the model parameters allows one to quantitatively compare the extraction efficiencies under various extracting conditions. Based on the results, we recommend the original sample should be diluted with the volume ratio of above 1 : 2 and a raw sample should be treated quickly to prevent the reduction of sample homogeneity and original integrity.

## 1. Introduction

EPSs are the important constituents of microbial aggregates such as activated sludge flocs and biofilms [1–3]. Many studies highlighted several aspects of EPS including their effects on flocculation, settling, dewatering of activated sludge, and also their spectral features and size distributions [4–7]. The detrimental effects of EPS on membrane filtration have been recognized for a long time particularly in terms of the performance of membrane bioreactors [8–11]. Although EPS content represents an essential element of biomass, the extraction methods are not fully standardized to date [12]. Several methods have been proposed to extract EPS from various microbial systems for further analysis of the liquid phase extract. Unfortunately, however, the individual methods result in inconsistent efficiencies, variable yields, and quality of EPS even for the same biomass sample [13, 14]. Prior studies demonstrated that a physical extraction method based on cation exchange resin successfully provides a high EPS recovery rate and the least contamination and cell lysis [7, 15].

The recovery rate and the composition of EPS appear to be strongly affected by the protocol of the extraction method [14]. As several other researchers have demonstrated, four major factors are typically associated with the yield efficiency of EPS extraction, which include the resin dose, the extraction time, the intensity of stirring, and the sample volume (i.e., sample dilution). For example, the extraction yield of EPS tends to increase with a higher dose of the resin up to 60–80 g/gVSS [16]. The amount of EPS extracted also increases with the extraction time, and it may gradually reach a certain level representing a maximum amount of extractable EPS. The extraction time may range from 1 to 17 hours, but extraction time over 12 hours is, generally, accepted as a standard, in most cases, to achieve a maximum amount (Table 1). However, it should be noted that the resin dose and the extraction time used in the previous studies were arbitrarily decided based on personal experiences, which did not account for the inherent process kinetics. 

The effects of sample preparation such as sample dilution and storage have not been well documented either in many prior EPS studies. Sample dilution is often required when an insufficient amount of sample is harvested or a relatively high dose of resin is needed for EPS extraction from suspended solids with a high concentration. It is often assumed that the maximum amount of extracted EPS does not depend on the sample volume. However, many characteristics of EPS extracted from sludge may vary with sample dilution if the heterogeneity of the sludge cannot be neglected. Potential changes in the integrity of the raw sludge sample can be of another concern, which may lead to the variation in the extraction yield of the EPS. It is necessary to examine the impacts of the raw sample's integrity on the EPS extraction in terms of the storage time and the condition. 

The main objective of this study was to analyze the extraction kinetics of EPS in terms of the extraction time at different resin doses. From this work, we derived a simple empirical formula on the extraction kinetics, that enables one to evaluate the extraction efficiencies at different experimental conditions. We also examined here the effects of other experimental conditions such as sample dilution and storage time of the sample on the yield of the EPS extraction. 

## 2. Materials and Methods

### 2.1. Source of Activated Sludge

Activated sludge samples were obtained from a municipal wastewater treatment plant (Gwachon, Korea). This plant employs a conventional activated sludge process, operated with 6–8 hours of hydraulic retention time and 8.7 m^3^/day of excess sludge production, to treat an average of 22,000 m^3^/d of domestic wastewater. Typical removal rates of biochemical oxygen demand (BOD), chemical oxygen demand (COD), suspended solids (SS), total nitrogen (TN), and total phosphorus (TP) correspond to 97%, 94%, 97%, 54%, and 76%, respectively. The samples used in this study were taken from a line returning the sludge from final sedimentation to the aeration reactor.

### 2.2. Extraction Procedure and EPS Analysis

EPS extraction was performed using cation resin as described by previous studies [7, 16]. with the following protocol. The CER used here was DOWEX 50 × 8, 20–50 mesh in the sodium form (Aldrich 42878-7). The CER was washed for 1 hour in an extraction buffer solution (Na_3_PO_4_ 2 mM, NaH_2_PO_4_ 4 mM, NaCl 9 mM, KCl 1 mM, pH 7) to remove any impurities. Before extraction, activated sludge was allowed to settle for 2 hours at 4°C and the supernatant was discarded. The settled sludge was centrifuged at 3,000 RPM for 5 minutes at 4°C and the supernatant was decanted again. The sludge pellets were resuspended in the buffer solution, and after one-hour settling, the supernatant was discarded to remove the dissolved organic carbon (DOC) in the solution. Buffer solution was added again to the settled sludge to make up the original volume. Volatile suspended solids (VSSs) of this sludge sample were measured. CER was then added to the sludge, and the mixture was stirred for different times at 4°C. After the stirring was finished, the supernatant with the extracted EPS was harvested by centrifugation for 1 minute at 12,000 RPM at 4°C. The collected supernatant was centrifuged again for 15 minutes at the same RPM at 4°C in order to completely remove any remaining solids. The extracted EPS was finally quantified by measuring DOC using a TOC Analyzer (*VCPN*-6000, Shimadzu, Japan).

### 2.3. Experimental Conditions

Three different doses (30, 70, and 100 g/g VS) of CER were taken into account for the experiments to evaluate the effect of CER dose. All mixtures were stirred at 600 RPM for 16 hours, and samples were collected at different times of 0, 0.5, 1.0, 2.0, 4.0, 8.0, and 15.0 hours. The EPS content of each sample was measured as described previously. The EPS extraction was conducted using the original and the diluted samples. The raw activated sludge was diluted with deionized distilled water (DDW), and the EPS content of the diluted sample was compared with the original sample (i.e., undiluted raw activated sludge). To test the effects of sample storage, the activated sludge was washed with a buffer solution and stored in a refrigerator at 4°C without extraction. After different storage times of 24, 48, 72, and 144 hours, EPS extraction was performed using the stored samples. Immediately before extraction, DOC concentration in the supernatant of the stored raw sludge sample was measured to account for DOC release from the solids during the storage. In addition, a solids-free EPS solution extracted from the sludge was stored as well, and its concentration was measured after the storage times of 24, 48, 72, and 144 hours.

## 3. Results and Discussion

### 3.1. Kinetics of Extraction Yield for Different Contact Times

**Figure 1:**
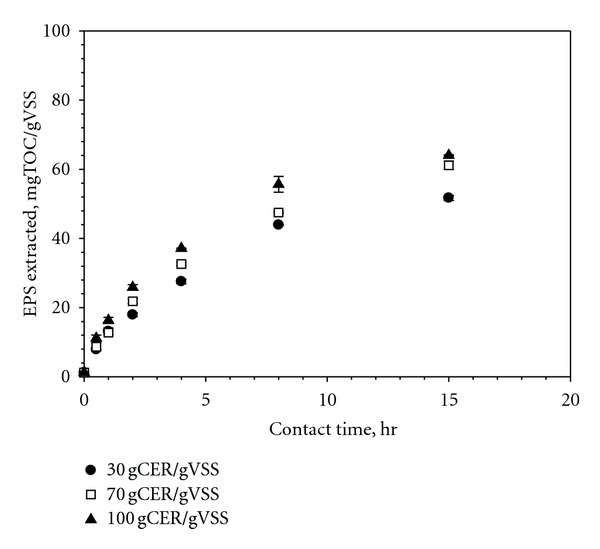
Effect of contact time and CER dose on EPS extraction yield (stirring intensity = 600 RPM).

Figure 1 presents changes of extracted EPS concentrations with contact times at different CER doses. The extraction yield increased with the contact time. The kinetics of the EPS yield appears rather dynamic, exhibiting a sharp increase at initial contact times followed by a weak enhancement with time. For example, the extraction yield at a contact time of 4 hours was nearly half of the yield at 15 hours. Our results clearly demonstrated that the quantity of the extracted EPS is strongly influenced by the specifics of the experimental protocol (contact time for this case). Although the kinetics of the extraction may vary from sample to sample, the maximum extractable EPS concentration (i.e., *C*_ max _) is of common concern to EPS research. 

**Figure 2:**
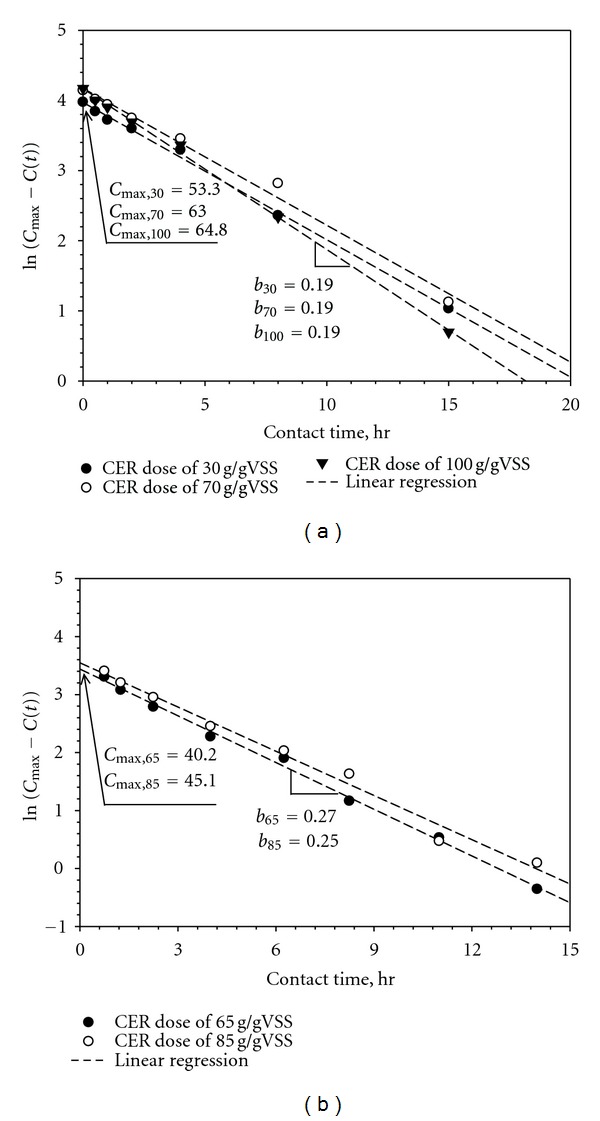
(a) Semilog plots of contact time *t* and ln⁡[*C*_ max_ − *C*(*t*)] to determine the parameters, *b* and *C*_max_. (b) Application of (1) and the linear regression of the data referred from Frølund et al. 1995. [18, Figure 1, page 1753].

For the successful estimation of the maximum concentration, a simple empirical model was proposed in this study. The shape of the extraction kinetics in Figure 1 suggests potential existence of pseudo-first-order kinetics with respect to the difference between *C*_ max_ and the actual concentration, *C*(*t*). When the data from Figure 1 was plotted in a semilog scale, a linear relationship, expressed by the following empirical function, was established (Figure 2(a)):

(1) $$\begin{array}{c} \ln\left[C_{\text{ max }}-C\left(t\right)\right]=-bt+K. \end{array}$$

Since *C*(*t* = 0) approaches zero when the background DOC is subtracted, the value of *K* is related to *C*_ max_ (i.e., *K* = ln⁡(*C*_max_)). Therefore, (1) can be arranged as follows:

(2) $$\begin{array}{c} C\left(t\right)=C_{\text{max}}\left[1-\exp\left(-bt\right)\right]. \end{array}$$

The parameter *b* describes the kinetics of the extraction process and it defines the half saturation time, *T*_1/2_ to reach the maximum EPS concentration:

(3) C(T1/2)=Cmax2=Cmax[1−exp⁡(−b·T1/2)],T1/2=b−1ln⁡⁡(2).

The CER process can be quantitatively analyzed by determining the two parameters, *C*_ max_ and *b*. The unknown parameters can also be obtained by the best fit of nonlinear regression using statistic tools. Figure 2(a) presents the results of this study with the best estimates of the corresponding *b* and *C*_ max _values.

The estimated values of *C*_ max_ for different CER doses indicate that the 30 gCER/gVSS dose was not sufficient to recover all possible extractable EPS from the activated sludge sample used for this study (Table 2). The value of the kinetic parameter, *b*, for the 30 and 70 g dose was almost identical (0.16 and 0.17 hr^−1^, resp.). The CER dose of 100 g gave a slightly higher *b* value of 0.19 hr^−1^ compared to the lower CER doses. As the CER dose increased to 233%, *T*_1/2_ decreased only by 17%, that corresponds to 3.71 and 3.07 hours for 30 g and 100 g of CER doses, respectively. Our result suggests that the half saturation time may not be strongly affected by the experimental condition of CER dose. Our proposed empirical model was applied to the data produced by another investigator [14] (Figure 2(b); Table 2) as well, in which EPS extraction was conducted at the stirring intensity of 900 RPM and the CER doses of 65 and 85 g/gVSS. Despite the fact that the extracted EPS was quantified by carbohydrate content for the study, the data appear to be successfully described by the simple empirical model (Figure 2(b)).

Our proposed approach based on the empirical model offers two significant advantages. First, the evaluation of EPS extraction yield with respect to its kinetics is allowed to estimate the maximum extractable EPS concentration, which is an intrinsic feature of the examined sludge. Second, the approach helps the reduction of the time required for the whole extraction process. Although only two data points are necessary to estimate the kinetic parameters of *b* and *C*_ max _, the quality of the estimates would be improved with the additional number of measurements. It should be noted that the mathematical form of (1) is not based on any theoretical consideration but it simply reflects the changes of the observed EPS concentrations with contact time.

### 3.2. Sample Dilution

**Figure 3:**
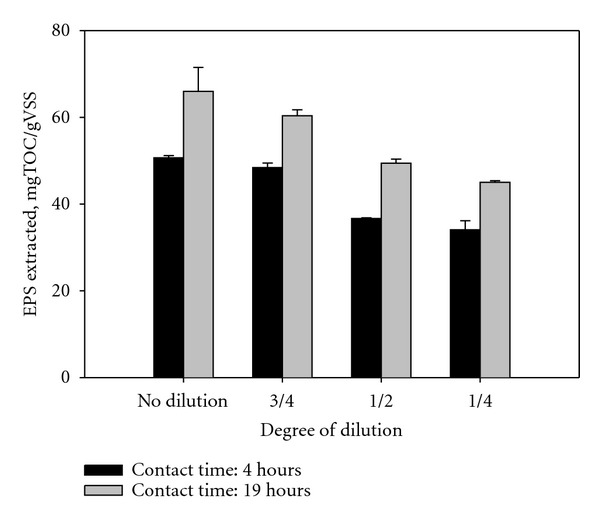
Effect of sample dilution on the EPS extraction (resin dose = 70 g/gVSS; stirring intensity = 600 RPM).

For the evaluation of sample dilution effect, EPSs were extracted at two different contact times from a sludge sample using various dilution factors, in which the original raw sample was diluted by DDW with the volume ratios of 3 : 4, 1 : 2, and 1 : 4. Irrespective of the contact time, the extracted EPS tends to decrease with increasing dilution (Figure 3). As presented in Table 3, the estimates of *b* and *C*_ max_ for the cases of the dilution obtained indicate that as the dilution degree increased, *C*_ max _decreased while *b* and *T*_1/2_ did not change. The results suggest that the sample dilution should be avoided for maximum extraction of the EPS. For the dilution of 1 : 2 and 3 : 4, however, the extraction yields remained at at least 70% of the amount of the EPS extracted from the nondiluted sample. Thus, we propose that the original sample should be diluted with the volume ratio of below 1 : 2 when the sample dilution is inevitable. The changes in *C*_ max_ values with the sample dilution may be attributed to the heterogeneity within the sample caused by different physicochemical properties of the individual activated sludge. For example, the content of total EPS released tends to increase linearly as the dispersed concentration of sludge increased under the same extraction condition [20]. In addition, EPS content may differ by flocs, and even floc size may not be the same within the sample. A previous study showed that the amount of EPS extracted was significantly decreased along with the increasing floc size ranging from 0.18 to 0.9 mm [21]. In this case, the integrity of the sample may decrease by the dilution, not fully reflecting the exact characteristic of the original whole sample.

### 3.3. Sample Storage

**Figure 4:**
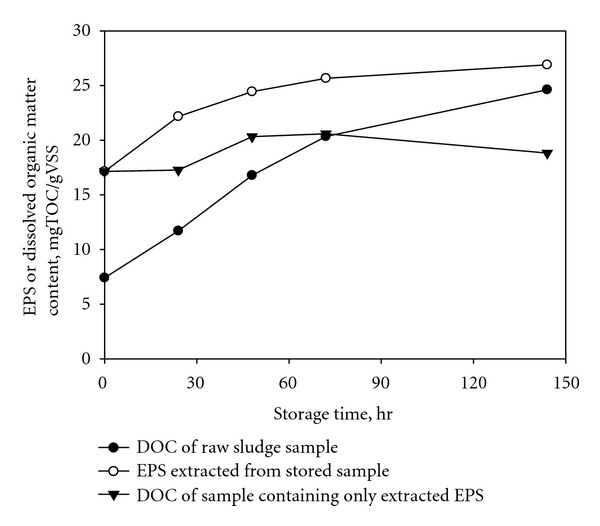
Storage effect on the extraction process (Resin dose = 70 g/gVSS; contact time = 1.5 hours; stirring intensity = 900 RPM). All the samples were stored at 4°C.

Figure 4 showed the changes of EPS or DOC concentrations with the storage time of the sample up to 144 hours. The sample containing extracted EPS only did not exhibit any consistent trend with the storage time in the DOC concentration, ranging from 17 to 21 mgC/gVSS. The average value was 18.8 ± 1.6 mgC/gVSS. Once EPS was extracted from the sludge sample, however, the EPS appears to keep its original quantity under storage at 4°C. In contrast, the content of EPS extracted from the stored raw samples showed an increasing trend with the storage time. The content finally reached 60% higher than the initial value (from 17.1 to 26.9 mgC/gVSS) after 144 hours of storage time. Because a washing step prior to the EPS extraction sufficiently removed the DOC of the residual supernatant of the stored sludge, our result clearly indicates the increase of the EPS content per unit biomass with the duration of storage. The DOC concentration in the stored sludge sample, which was measured after centrifugation, also increased from 7.4 to 24.6 mgC/gVSS. This observation is probably attributed to the cell lysis during the storage or the dismantlement of floc followed by the release of organic matters from the raw sludge sample. Our results imply that the EPS extraction should be performed as quickly as possible to obtain the representative EPS content of a raw sludge sample.

## 4. Conclusions

In this paper, a simple empirical model was developed for evaluating the EPS extraction using cation exchange resin. This tool included a formula describing the EPS extraction kinetics, which is allowed to predict the maximum extractable EPS (*C*_ max _) and to calculate the extraction speed or the efficiency, and the half saturation time (*T*_1/2_). The proposed empirical model was based on the recognition of the extraction as a dynamic process. It can help to substantially reduce the whole experimental time. Using the model also makes it possible to design the optimized extraction process by comparing quantitatively the extraction results for different experimental conditions. The yield of EPS extraction tends to increase with the extraction time and the CER dose. However, the maximum amount of EPS extraction was present for the different experimental conditions. The maximum yield of EPS extraction was influenced by the sample dilution as well, exhibiting a decreasing trend with increasing dilution factor. The amount of EPS extracted from a raw sample depended on the storage time. Once EPS was extracted from the sludge sample, however, the EPS appears to keep its original quantity under the storage at 4°C.

## Figures and Tables

**Table 1:** Different experimental conditions applied for EPS extraction in the cation exchange resin method.

	Extraction time	Stirring intensity	CER dose^∗^	Sample volume
Frølund et al. [16]	17 hrs at 4^°^C	900 RPM	65–85 g/gVSS	300 mL sludge to 500 mL total vol.
Domínguez et al. [7]	16 hrs at 4^°^C	900 RPM	70 g/gVSS	150 mL sludge^∗∗^
Nielsen et al. [17]	12 hrs at 4^°^C	900 RPM	70–75 g/gVSS	200 mL sludge^∗∗^
Dignac et al. [18]	1 hr at 4^°^C	Not specified	60 g (in 100 mL sludge)	100 mL sludge^∗∗^

*Cation exchange ion used; DOWEX 50X8, 20–50 mesh (Fluka 44445).

^∗∗^Total volume is not specified.

**Table 2:** Estimated kinetic parameters (*b*, *C*_max⁡_, *T*_1/2_) based on the best fit of the empirical model for different resin doses. The stirring intensity is 600 RPM.

CER doses	Kinetic parameters	Estimated values	*R* ^2^ values	
	*b* (hr^-1^)	0.17 ± 0.02		
30 g/gVSS	*C*_max⁡_ (g/gVSS)	53.29 ± 2.74	0.9974	
	*T*_1/2_ (hour)	3.71		
	*b*	0.16 ± 0.017		This study
70 g/gVSS	*C*_max⁡_	63.10 ± 2.47	0.9900	
	*T*_1/2_	3.99		
	*b*	0.19 ± 0.023		
100 g/gVSS	*C*_max⁡_	64.80 ± 3.81	0.9982	
	*T*_1/2_	3.07		

	*b* (hr^-1^)	0.27		
65 g/gVSS	*C*_max⁡_ (g/gVSS)	40.2	0.9963	
	*T*_1/2_ (hour)	2.8		Frølund et al. [19]
	*b*	0.25		
85 g/gVSS	*C*_max⁡_	45.1	0.9871	
	*T*_1/2_	2.6		

**Table 3:** Estimation of *C*_max⁡_, *b*, and *T*_1/2_ using (2) and (3) for the dilution of raw sludge sample.

Dilution	1: 4	1: 2	3: 4	1: 1 (No dilution)
*C*_max⁡_ (mgTOC/gVSS)	41.75	46.19	57.06	62.74
*b* (hr^-1^)	0.33	0.32	0.39	0.35
*T*_1/2_ (hr)	2.10	2.17	1.78	1.98
